# Effects of Working Memory, Strategy Use, and Single-Step Mental Addition on Multi-Step Mental Addition in Chinese Elementary Students

**DOI:** 10.3389/fpsyg.2019.00148

**Published:** 2019-02-05

**Authors:** Yi Ding, Ru-De Liu, Hongyun Liu, Jia Wang, Rui Zhen, Rong-Huan Jiang

**Affiliations:** ^1^Graduate School of Education, Fordham University, New York City, NY, United States; ^2^Institute of Developmental Psychology, Beijing Key Laboratory of Applied Experimental Psychology, National Demonstration Center for Experimental Psychology Education, Faculty of Psychology, Beijing Normal University, Beijing, China; ^3^Teachers’ College, Beijing Union University, Beijing, China; ^4^Institute of Psychological Sciences, Hangzhou Normal University, Hangzhou, China

**Keywords:** working memory, automaticity, strategy use, mental addition, Chinese elementary students

## Abstract

The aim of this paper was to examine the roles of working memory, single-step mental addition skills, and strategy use in multi-step mental addition in two independent samples of Chinese elementary students through different approaches to manipulate two dimensions of task characteristics (the primary task). In Study 1, we manipulated strategy types through the dimension of schema automaticity (whether intermediate sums were 10s) and the dimension of working memory load (WML, two steps versus four steps). A hierarchical linear model (HLM) analysis was conducted at case level, strategy level, and individual level. In Study 2, we manipulated task characteristics through schema automaticity (one-time versus two-time regrouping) and the WML (partial versus complete decomposition). A three-level HLM analysis was applied. The general findings of Study 1 and Study 2 suggested that shorter response time on single-step mental addition corresponded to shorter response time on multi-step mental addition. The use of strategies (from easier to more difficult strategies) negatively predicted response time on multi-step mental addition. Easier strategy was associated with shorter response time on multi-step mental addition. Better phonological loop was associated with shorter response time on multi-step mental addition. The findings in both studies highlighted the important role of phonological loop in mental addition in Chinese children, suggesting that the involvement of a specific subcomponent of working memory in mental arithmetic might be subject to linguistic, instructional, and contextual factors.

## Introduction

Research in mental arithmetic has received increasing attention in the past four decades (e.g., Groen and Parkman, 1972; Ashcraft, 1992, 1995; Sowder, 1992; Carroll, 1996; LeFevre et al., 2003; Liu et al., 2015). Mental arithmetic refers to the process of performing arithmetical calculation in the mind without external support such as using paper and pencil, calculators, or computers (Reys, 1984; Maclellan, 2001). Within basic arithmetic operations of addition, subtraction, multiplication, and division, addition is often learned more easily in children’s learning trajectory, and addition serves as the foundation for learning the other three operations (Beishuizen et al., 1997; Bryant et al., 1999; Torbeyns et al., 2009). In the domain of mental addition, researchers often explored simple (single-digit) mental addition and factors that affected simple mental addition in adult learners such as college students (LeFevre et al., 1996; Butterworth et al., 2001; De Rammelaere et al., 2001; Hecht, 2002). In recent years, more attention has addressed complex mental addition (e.g., addition involving two or more digits); however, the participants have been predominantly adult learners (Green et al., 2007; Imbo and LeFevre, 2009; Klein et al., 2010; Moeller et al., 2011).

Mental addition can be affected by individual characteristics such as experiences in arithmetic problem solving, working memory capacities, age, schema automaticity obtained by each individual, and strategy used for problem solving (Zbrodoff and Logan, 1986; Geary et al., 2004; Tronsky, 2005; Imbo et al., 2007; Arnaud et al., 2008). Mental addition can also be affected by task characteristics such as the difficulty level of the presented problems, types of problems (e.g., addition, subtraction, multiplication, and division), practice effects, and working memory load (WML) required by the tasks (DeStefano and LeFevre, 2004; Kalaman and LeFevre, 2007; Imbo and Vandierendonck, 2008). In our previous studies, we examined the effects of simple mental addition on complex mental addition, the effects of subcomponents of working memory on complex mental addition, and the moderating effects of working memory on single-step mental addition in relation to multi-step mental addition (Ding et al., 2017; Liu et al., 2017; Ding et al. unpublished). For our current studies, we recruited Chinese elementary students (Chinese children are anticipated to achieve a high level of proficiency of basic arithmetics in the early years of elementary school; People’s Education Press, 2017) and focused on complex mental addition to explore the roles of working memory, single-step mental addition, and strategy use (manipulated by schema automaticity and WML) in multi-step mental addition.

### Working Memory and Mental Arithmetic

Although children might activate different strategies for addition and multiplication, it is generally believed that children tend to be slower and make more errors with larger problems (the problem-size effect) and with problems that require carrying (DeStefano and LeFevre, 2004). If a certain amount of working memory is required for calculation of single-digit problems, we anticipate that increased working memory would be required for calculations involving multi-digit problems. Thus, in the following review of literature, we summarized findings according to single-digit problems and multi-digit problems, rather than types of calculation (i.e., addition versus multiplication).

Mental arithmetic involves encoding the presented information, executing the calculation in the mind, and providing a response (LeFevre et al., 2005). During the calculation process, one must temporarily maintain the intermediate results while continuing the calculation in order to reach the final solution. The role of working memory in mental arithmetic has been examined in empirical studies (e.g., Lemaire et al., 1996; Campbell, 1999; Lee and Kang, 2002; DeStefano and LeFevre, 2004; Meyer et al., 2010; Friso-van den Bos et al., 2013). Based on Baddeley’s (1992) model of working memory, many researchers explored phonological loop, visuospatial sketchpad (VSSP), and central executive as the subcomponents of working memory in relation to mental arithmetic. However, the findings regarding the involvement of subcomponents of working memory in mental arithmetic have been quite mixed (DeStefano and LeFevre, 2004; Meyer et al., 2010; Caviola et al., 2012; Friso-van den Bos et al., 2013).

The phonological loop was found to be involved in the process of maintaining intermediate sums during multi-digit mental addition (Ashcraft and Kirk, 2001; Noël et al., 2001; DeStefano and LeFevre, 2004). Seitz and Schumann-Hengsteler (2000, 2002) found that maintaining intermediate results requires the involvement of both the central executive and the phonological loop, and they reported the involvement of the phonological loop on two-digit plus two-digit addition tasks. Heightened phonological loop skills appear to facilitate performance in complex mental addition, indicating that strong phonological loop is associated with shorter response time (Fürst and Hitch, 2000; Trbovich and LeFevre, 2003; Caviola et al., 2012).

The findings regarding the VSSP in mental arithmetic are mixed, although there was some evidence to suggest that VSSP might be involved in multi-digit problems (e.g., Logie et al., 1994; Lee and Kang, 2002; Trbovich and LeFevre, 2003; Ashkenazi et al., 2013; Laski et al., 2013). However, some studies reported null findings regarding the role of VSSP in multi-step mental arithmetic (Noël et al., 2001; Liu et al., 2017). Some reported that the impact of VSSP on mental arithmetic decreased as children matured (McKenzie et al., 2003; Holmes et al., 2008). In short, the role of the VSSP in multi-step mental arithmetic remains uncertain and warrants further research (DeStefano and LeFevre, 2004). The findings were not sufficiently comprehensive to draw a conclusion.

The central executive is responsible for planning, manipulating, and sequencing of information. The central executive also coordinates the activities of phonological loop and the VSSP. The findings regarding the central executive in mental arithmetic are inconsistent. In terms of single-digit arithmetic, some evidence pinpointed that central executive resources are required to process single-digit problems (Kaye et al., 1989; Ashcraft et al., 1992; Lemaire et al., 1996; De Rammelaere et al., 1999, 2001; Seitz and Schumann-Hengsteler, 2000, 2002; Hecht, 2002). In multi-digit arithmetic, there has been evidence for the involvement of the central executive in maintaining intermediate results during calculation (Heathcote, 1994; Logie et al., 1994; Fürst and Hitch, 2000; Seitz and Schumann-Hengsteler, 2000, 2002), whereas the influence of updating (one component of the central executive) was not significant (Liu et al., 2017).

In short, there were relevant consistent findings regarding the role of the phonological loop, rather than the VSSP and the central executive system, in mental arithmetic. In addition, because the Chinese mathematics curriculum emphasizes rote memorization, drills, and practices to enhance proficiency in mental arithmetic, the phonological loop appeared to be more relevant to the instructional and linguistic contexts in which Chinese children learn mental arithmetic. In Liu et al. (2017), Chinese children’s accuracy and response time on mental multiplication were most susceptible to phonological loop influence, when phonological loop, VSSP, and central executive tasks were tested. Thus, our examination of working memory focused on the phonological loop in the present studies.

### Direct Retrieval and Schema Automaticity in Relation to Mental Arithmetic

Many factors contribute to how quickly and accurately an individual can execute mental arithmetic. One general factor is the individual’s ability to understand and apply problem-solving strategies. Research has shown that it takes a long time for most students to transition from a direct modeling of the problem context, counting all of the numbers one by one, to the point that they can use direct retrieval of math facts (Mulligan and Michelmore, 1997; Downton, 2008). In comparison to low-achieving students, Zhang et al. (2014) found that high-achieving students demonstrated greater strategy flexibility during problem solving and were more accurate in direct retrieval and performing mathematics algorithm strategies. Direct retrieval is an important component of the elementary mathematics curriculum. Direct retrieval of number combinations is often achieved by typically developing students by the beginning of third grade (e.g., approximately 8 to 9 years old) in the United States (Miller and Hudson, 2007). Through repetitive practice, students learn to directly retrieve mathematics facts. Although direct retrieval is listed as one of the strategies for problem solving, it involves the retrieval of mathematics facts from long-term memory (i.e., the answer is obtained immediately) and does not involve the process of using multiple steps for problem solving.

According to the cognitive load theory (Sweller, 1988; Paas et al., 2003; van Merriënboer and Sweller, 2005), human beings have limited working memory to deal with all conscious activities and unlimited long-term memory to store facts and schemas. When students achieve automaticity with mathematic facts, they have attained a level of mastery that enables them to retrieve those facts from long-term memory without conscious effort or attention, which reflects a highly efficient process (Ponser and Snyder, 1975). A schema can be considered as a single entity that comprises multiple elements and allows humans to bypass irrelevant details. Automaticity is an important component in the process of forming schema and is often achieved after practice. In the domain of mathematics operation, an individual who has attained the level of automaticity can directly retrieve facts from long-term memory without conscious cognitive processing, which is considered direct retrieval (Siegler and Shrager, 1984; Siegler and Jenkins, 1989; Shrager and Siegler, 1998; Geary, 2011). When multiple elements of basic arithmetic facts form large operation units, students reach the level of mastery of schema automaticity after repeated practice and frequent exposure to the tasks (Sweller, 1988; Logan and Klapp, 1991; Wilkins and Rawson, 2011). For example, when a student encounters 25 × 6 the first time, he or she might use a regular algorithm to obtain the result. However, after repeated practice, the student might memorize the result and directly retrieve the mathematics fact (Compton and Logan, 1991; Wilkins and Rawson, 2011) without utilizing regular operations or complex strategies.

Liu et al. (2017) reported that Chinese school systems predominantly emphasize rote memorization of single-digit and two-digit arithmetic facts. Because of repeated practice, many Chinese elementary students eventually reach the level of mastery of direct retrieval of basic arithmetic facts. Many Chinese elementary students not only retrieve basic arithmetic facts, but also rote memorize many schemas such as 25 × 4 and 17 + 13. In single-step mental addition, Chinese elementary students utilize direct retrieval and schemas that become automatic. Thus, direct retrieval and schema automaticity in single-step mental addition might have an impact on the response time and accuracy rate of multi-step mental addition.

### Strategy in Relation to Complex Mental Arithmetic

Children attempt different strategies such as decomposing and transformation when they solve complex arithmetic problems (Ashcraft and Fierman, 1982; Beishuizen et al., 1997; Lucangeli et al., 2003; Arnaud et al., 2008; Lemaire and Callies, 2009). For example, children could decompose “45 + 39” into “45 + 40 - 1,” “40 + 40 + 5 - 1,” “40 + 30 + 5 + 9,” “45 + 30 + 9,” or “50 + 34.” When children apply different strategies during mental arithmetic, they might utilize some schema such as “40 + 30” or “45 + 30” and need to activate working memory to complete processes such as transformation, temporarily memorizing intermediate sums, and operation.

When children process complex mental arithmetic, they use different strategies that are associated with different levels of schema automaticity and WML. Given an arithmetic problem (e.g., 16 + 27), a student could use complete decomposition (e.g., decomposing 16 + 27 to 10 + 6 + 20 + 7, three steps in total) to carry out the calculation step by step. Step-by-step full decomposition or the use of an arithmetic algorithm often involves many steps requiring a large amount of working memory resources, which in turn might increase the response time to obtain a solution. In contrast, a student could use an automatized schema (e.g., converting 16 + 27 to 16 + 24 + 3 = 40 + 3 = 43, two steps in total) that leads to fewer steps (requiring fewer working memory resources) and shorter response time, in comparison to full decomposition or the use of an arithmetic algorithm. As a result, the effectiveness of a strategy used for mental arithmetic might be contingent upon the automaticity level of the strategy that was retrieved and the WML involved during problem solving.

In a previous study, we examined schema automaticity and WML through the perspective of task characteristics (Ding et al., 2017) and manipulated the levels of schema automaticity and WML (i.e., the original problem was 8 + 18 = 26). Schema automaticity was operationalized by having the intermediate sum being 10 or the intermediate sum not being 10. In terms of WML, it was operationalized in the way that the problem had fewer versus more steps. There were four strategy conditions: (a) problems with high schema automaticity and low WML (8 + 12 + 6 =), (b) problems with high schema automaticity and high WML (8 + 2 + 7 + 3 + 6 =), (c) problems with low schema automaticity and low WML (8 + 6 + 12 =), and (d) problems with low schema automaticity and high WML (8 + 6 + 3 + 7 + 2 =). We were able to find significant main effects of schema automaticity and WML and a significant interaction effect between these two factors in mental multiplication and addition among Chinese elementary students. Our findings supported the important roles of schema automaticity and WML during mental arithmetic.

Because this study focused on Chinese children, it is important to have a brief review of how Chinese children learn math. According to Wei (2014), math education in China has a number of unique characteristics. Chinese children start learning the mathematics facts at a very young age (age 4 or 5 years through informal family education). According to People’s Education Press, 2017, addition and subtraction of single-digit numbers should be mastered with high fluency by the end of first grade (age 6). Multiplication is introduced in the fall semester of second grade (age 7) and should be mastered by the end of second grade (People’s Education Press, 2017). Most simple arithmetic facts such as addition, subtraction, multiplication, and division are taught through memorization and routine practice (People’s Education Press, 2017). Children take at least one math class (40 min) with a single-subject math teacher (i.e., math teachers teach math classes in multiple classrooms at the same grade) each day, with at least 30 min of math homework on a daily basis. One main goal of China’s math education is to develop not only conceptual understanding (what), but also procedural knowledge (how to) through practice and application (People’s Education Press, 2017). Accuracy and fluency are highly regarded. From Chinese math teachers’ standpoints, knowing a math concept (knowing the concept) without the abilities to efficiently solve the math problem (executing the operations) does not indicate skill acquisition. Thus, Chinese children are expected to have a very high level of accuracy and fluency on basic math facts. Given the structure of Chinese math education, automaticity and working memory appear to play a critical role in children’s learning.

### The Purpose of the Present Study

In Ding et al. (2017), we found significant main effects of schema automaticity and WML in relation to mental multiplication through the perspective of task characteristics (examining how the same group of students responded differently to different strategy conditions). In Liu et al. (2017), our findings indicated the important role of the phonological loop in mental multiplication through the perspective of individual characteristics (examining how individuals’ subcomponents of working memory affected mental multiplication). Similar findings were revealed in our study regarding mental addition in Chinese children (2018). In short, the effectiveness of mental arithmetic is contingent upon an individual’s basic mental addition skills, the strategy selected, and the working memory involved during problem solving. The purpose of this study was to examine the effects of single-step mental addition skill, strategy use, and working memory on multi-step mental addition.

Previous studies often examined the effects of simple mental arithmetic skill, strategy use, and working memory on complex mental arithmetic in isolation. We extended the previous studies in four ways. First, we simultaneously examined the effects of single-step mental addition, strategy conditions, and working memory on multi-step mental addition. Second, we manipulated the strategy through two dimensions of task characteristics, including schema automaticity and WML, to control the difficulty levels of strategy conditions. Thus, we generated four strategy conditions. We utilized the no-choice format based on Siegler and Lemaire (1997) in order to require all participants to execute the four strategies to examine how the difficulty levels of strategy use affected mental addition and this approach was validated in Ding et al. (2017). Third, we used a three-level hierarchical linear model (HLM) analysis to examine the relations of key variables at the student level, strategy level, and item level. Fourth, we tested our research questions in two studies. In Study 1 and Study 2, we used different approaches to decompose the addition problems and used different approaches to manipulate the levels of schema automaticity and WML. We wanted to explore whether Study 1 and Study 2 both supported the effects of single-step mental addition skill, strategy conditions, and working memory on multi-step mental addition. Based on the findings of Ding et al. (2017), Liu et al. (2017), Ding et al. (unpublished), we anticipated that better single-step mental addition performance would be associated with better multi-step mental addition performance (Hypothesis 1); the strategy with high schema automaticity and low WML would be associated with shorter response time on multi-step mental addition (Hypothesis 2); and better working memory capacity would be associated with shorter response time and higher accuracy rate on multi-step mental addition (Hypothesis 3) in both Study 1 and Study 2.

## Study 1

### Design

The dependent variable was the response time of the multi-step mental additions. The independent variables included the response time of the single-step mental additions, strategy conditions (we manipulated the levels of schema automaticity and WML to reflect four strategy conditions), and the phonological loop task. We considered the single-step mental addition performance as an indicator of children’s basic mental addition skills. We considered the multi-step mental addition performance as an indicator of children’s skills on complex mental addition.

To account for student-level, strategy-level, and item-level variances, a three-level HLM analysis was applied. At the item level (Level 1), we used multi-step mental addition performance as the dependent variable and single-step mental addition performance as the independent variable to examine the effect of single-step mental addition on multi-step mental addition. At the strategy level (Level 2), we used the four strategy conditions as the independent variable and the intercept of Level 1 as the dependent variable to examine the effects of strategy use on multi-step mental addition. At the student level (Level 3), we used the phonological loop task as the independent variable and the intercept of Level 2 as the dependent variable to examine the effect of phonological loop on multi-step mental addition.

### Measures and Procedures

#### Strategy

In order to manipulate the levels of schema automaticity and WML to reflect the strategy used for each question, we alternated two aspects of the structural features of addition problems: WML was manipulated by the steps involved in operations (i.e., two steps versus four steps,) and schema automaticity was manipulated by whether the single-step addition involved intermediate sums of 10 (Lemaire and Callies, 2009; Klein et al., 2010). In teaching practice, students are often taught to add base 5 numbers such as 1 + 4, 2 + 3, and then base 10 numbers, such as 1 + 9, 2 + 8, 3 + 7, 4 + 6, and 5 + 5. In Chinese math curriculum, speeded arithmetic strategies are often taught to help students develop more efficient strategies and adding intermediate sums to base 10 is often utilized (e.g., transforming 7 + 9 + 13 to 7 + 13 + 9 = 20 + 9 = 29). Thus, in the present study, the problems with intermediate sums of 10 indicate a high level of schema automaticity, in comparison to problems without intermediate sums of 10. Given a problem such as 7 + 22 = 29, there were four strategy conditions: (1) problems with high schema automaticity and low WML such as 7 + 13 + 9 (there was one intermediate sum being 10 and there were two steps), (2) problems with high schema automaticity and high WML such as 7 + 3 + 4 + 6 + 9 (there were two intermediate sums being 10 and there were four steps), (3) problems with low schema automaticity and low WML such as 7 + 9 + 13 (there were no intermediate sums being 10 and there were two steps), and (4) problems with low schema automaticity and high WML such as 7 + 4 + 6 + 9 + 3 (there were no intermediate sums being 10 and there were four steps) (see Table 1). In order to ensure the participants would perform according to the imposed problem order and format, all problems were presented in the left-to-right order.

**Table 1:** Addition problems used during the testing (Study 1).

Original problems	High automaticity		Low automaticity	
	Low WML (1)	High WML (2)	Low WML (3)	High WML (4)
8 + 18 = 26	8 + 12 + 6 =	8 + 2 + 7 + 3 + 6 =	8 + 6 + 12 =	8 + 6 + 3 + 7 + 12 =
12 + 25 = 37	12 + 18 + 7 =	12 + 8 + 6 + 4 + 7 =	12 + 7 + 18 =	12 + 4 + 7 + 6 + 8 =
21 + 25 = 46	21 + 19 + 6 =	21 + 9 + 3 + 7 + 6 =	21 + 6 + 19 =	21 + 3 + 6 + 9 + 7 =
24 + 27 = 51	24 + 26 + 1 =	24 + 16 + 2 + 8 + 1 =	24 + 1 + 26 =	24 + 2 + 1 + 8 + 16 =
17 + 17 = 34	17 + 13 + 4 =	17 + 3 + 9 + 1 + 4 =	17 + 4 + 13 =	17 + 1 + 4 + 3 + 9 =
19 + 35 = 54	19 + 31 + 4 =	19 + 1 + 12 + 18 + 4 =	19 + 4 + 31 =	19 + 18 + 1 + 4 + 12 =
RT *M*/*SD* in seconds	3.48 (1.86)	5.28 (2.81)	6.48 (3.56)	9.03 (3.89)
Cronbach’s α for RT	0.81	0.68	0.71	0.85


*WML, working memory load; (1), (2), (3), and (4), conditions (1), (2), (3), and (4). RT, response time.*

Regression analysis treats all independent variables in the analysis as numerical, which means that these variables are interval or ratio scale variables. Our four strategy conditions were nominal scale variables that included four categories of strategies. Thus, dummy variables were created to correctly analyze categorical variables. First, we treated the strategy condition (1) as one category and the remaining three conditions as another category. Then, we had the coding for strategy-a (3, -1, -1, -1). Second, among the strategy conditions (2), (3), and (4), we treated the condition (2) as one category, and conditions (3) and (4) as another category. Then, we obtained strategy-b (0, 2, -1, -1). Finally, we compared conditions (3) and (4), so we obtained strategy-c (0, 0, 1, -1). We did not need a fourth dummy variable to represent condition (4) because all four strategy conditions were mutually exclusive (they did not overlap) and exhaustive (no other levels exited for this variable; Ding, 2000).

#### Multi-Step Addition Problems (Simultaneous Presentation)

In total, there were six original questions, and each original question was presented as four strategy conditions to reflect high or low schema automaticity and high or low WML. For example, an original problem was 12 + 25. There were four strategy conditions: (a) problems with high schema automaticity and low WML (12 + 18 + 7 =), (b) problems with high schema automaticity and high WML (12 + 8 + 6 + 4 + 7 =), (c) problems with low schema automaticity and low WML (12 + 7 + 18 =), and (d) problems with low schema automaticity and high WML (12 + 4 + 7 + 6 + 8 =). Thus, there were 24 multi-step addition problems. E-prime was used for programming. All problems were randomly presented by computers to counter the order effect. Prior to testing, a stimulus of “+” appeared in the center of the computer screen for 150 ms. The performance on addition problems measured by simultaneous presentation indicated student performance on multi-step mental addition. The participants were instructed to orally report the answer as soon as possible. When the examinee orally reported the answer, the examiner entered the answer and clicked the “return” key. Then, a stimulus of “+” appeared in the center of the computer screen and the examinee moved on to the next testing item. The computer recorded the accuracy and response time (i.e., the duration was from the point of stimulus presentation to the point that the examiner hit the enter key) for each testing item (i.e., both correct or incorrect items). Cronbach’s α was 0.89 for response time and 0.69 for accuracy, which is acceptable (DeVellis, 1991).

#### Single-Step Addition Problems (Successive Presentation)

The same 24 addition problems were re-used. However, to obtain the accuracy and response time on single-step addition, the presentation of each testing item such as “8 + 6 + 3 + 7 + 2 =” was successive. In other words, the computer first presented the single step of “8 + 6 =.” The participant obtained the answer of “14” and pressed the “Enter” key. Then, the computer presented the next step “+3”; the participant obtained the answer of “17” and pressed the “Enter” key. When the examinee orally reported the answer, the examiner entered the answer and clicked the “return” key. Then, a stimulus of “+” appeared in the center of the computer screen and the examinee moved on to the next testing item. There were 24 items in total. The response time on single-step addition was defined as the total response time on each successively presented item divided by the steps involved in that item. All problems were randomly presented by the computer. The computer automatically recorded the accuracy and response time for each item. The internal consistency for this instrument ranged from 0.68 to 0.86.

#### Working Memory Measure (Phonological Loop Task)

Based on our previous study examining subcomponents of working memory among Chinese elementary students, only phonological loop played a significant role in mental arithmetic, whereas VSSP and central executive did not play a significant role (Liu et al., 2017). Thus, we only included phonological loop as a measure of relevant working memory in the present study. The phonological loop task was developed based on Grant and Dagenbach (2000) and Wang et al. (2008). In total, there were 50 equations. Ten groups of equations consisted of two independent sequences of three, four, five, six, and seven equations. Participants were asked to determine whether the presented equation was correct or incorrect while they tried to memorize the second number of the equation (e.g., 7–3 = 4). Both correct and incorrect answers were provided. The participants used either the “left” or the “right” button of the mouse to indicate “correct” or “incorrect.” Participants were exposed to each equation a maximum of 4,000 ms. If a participant did not respond within 4,000 ms, the next equation automatically appeared on the computer. After one group of equations were presented, the participants were asked to enter all of the second numbers of those equations in a row. The E-Prime program randomly presented all equations. The second number in two adjacent equations should not be the same, and the second number in each equation should not be the same as the correct answer for that equation. The scores ranged from 0 to 50. Higher scores indicated better phonological loop. The internal consistency for this instrument in this sample was 0.81.

To counter an order effect, all problems of each task were randomly presented by the computers. E-prime was used for programming. Prior to testing, the participants received training through practice items. The participants completed three tasks in a random order.

### Participants

Chinese elementary students master under-100 addition and subtraction with and without regrouping by fall semester of Grade 2. They learn under-100 multiplication and division by the end of Grade 2. Running a power analysis on a repeated measures ANOVA with four measures, a power of 0.80, an alpha level of 0.05, and a medium effect size (*f* = 0.25) requires a sample size of at least 24 (Faul et al., 2013). We recruited 40 participants for Study 1. Thus, we recruited 40 typically developing third graders who should have fluently mastered under-100 addition, subtraction, multiplication, and division by the time of testing. The average age for the participants was 8.56 years (*SD* = 0.89) and 22 were females and 18 were males. The participants were randomly recruited from an elementary school in China. This study was approved by the Research Ethics Committee of Beijing Normal University and the principals of the participating schools. Written and Informed consent was obtained from the parents/legal guardians of participants.

### Results and Discussion

In Study 1, the main goal was to examine how single-step mental addition, strategy use, and working memory affected multi-step mental addition. To account for student-level, strategy-level, and item-level variances, a three-level HLM analysis was applied. Chang (2003) described HLM as a “regression of regression.” The Level 3 sample size was 40, the Level 2 sample size was 160 (40 students completed four strategy conditions), and the Level 1 sample size was 960 (40 students completed all 24 items). We maintained four decimals in the HLM results because HLM results often carry very small but practically meaningful numerical values (Chang, 2003, 2004).

All of the participants had very high levels of accuracy (ranging from 84.17 to 95.71% among four conditions), and there was little variation among the participants (*M* = 91.5%, *SD* = 8.2%). Thus, the measure of accuracy was excluded as a variable for analysis. We only used participants’ correct response time for further analysis. The descriptive statistics of different variables are listed in Table 2.

**Table 2:** Descriptive statistics of response time at item-, strategy-, and student-level (Study 1).

Variables	*N*	Mean	*SD*	Min	Max
**Item-level (Level 1)**					
Multi-step RT	896	7.62	4.72	1.45	39.04
Single-step RT	896	3.69	1.85	0.86	19.59
**Strategy-level (Level 2)**					
Strategy-a	160				
Strategy-b	160				
Strategy-c	160				
**Student-level (Level 3)**					
Phono	40	36.05	8.53	10.00	47.00


*We only analyzed correct response time. RT, response time (measured in seconds). There were 24 testing items. The multi-step response time was calculated based on the response time on each testing item. The single-step response time was calculated based on the response time on each testing item divided by the presentation steps involved in that item. Phono, phonological loop.*

In Table 3, the dependent variable was the average response time of multi-step addition at Level 1 (item level). The independent variable was the average response time of single-step addition at Level 1, indicating the basic single-step addition skill. *γ_100_* (0.4620) was significant, which suggested that single-step response time (indicating automaticity) had an effect on multi-step response time in the positive direction. This suggested that shorter response time on single-step mental addition led to shorter response time on multi-step mental addition, supporting Hypothesis 1.

**Table 3:** Effects of automaticity, strategy, and phonological loop on response time: three-level regression coefficients (Study 1).

Fixed effect		Coefficient	*SE*	T-ratio	Approx. df	*P*
**Multi-step RT as the outcome measure**						
*Student-level (Level 3)*						
For INTRCPT1	π_0_					
INTRCPT2	β_00_					
INTRCPT3	γ_000_	9.6469	1.7847	5.41	38	<0.001
Phono	γ_001_	–0.1017	0.0440	–2.31	38	0.027
*Strategy-level (Level 2)*						
For STATEGY-A	β_01_					
INTRCPT3	γ_010_	–1.0303	0.0707	–14.58	117	<0.001
For STATEGY-B	β_02_					
INTRCPT3	γ_020_	–0.5679	0.1388	–4.09	117	<0.001
For STATEGY-C	β_03_					
INTRCPT3	γ_030_	–2.2791	0.2617	–8.71	117	<0.001
*Item-level (Level 1)*						
For ST-RT slope	π_1_					
INTRCPT2	β_10_					
INTRCPT3	γ_100_	0.4620	0.1136	4.07	695	<0.001


*We only analyzed correct response time. INTRCPT, intercept; Phono, phonological loop; RT, response time; ST-RT, single-step response time. It is common to retain four decimals for HLM results (Chang, 2003).*

At Level 2 (strategy-level), the dependent variable was the intercept of Level 1 (the response time of multi-step mental addition). Four strategy conditions were treated as dummy variables, including strategy-a, strategy-b, and strategy-c. *γ_010_* (-1.0303), *γ_020_* (-0.5679), and *γ_030_* (-2.2791) were all statistically significant, suggesting that strategy use had effects on multi-step response time in the negative direction. The higher the coding values for the strategies, the smaller the intercept. As we explained earlier, our dummy variables coding for strategy conditions included strategy-a (3, -1, -1, -1), strategy-b (0, 2, -1, -1), and strategy-c (0, 0, 1, -1). The values of coding of dummy variables followed a descending order from strategy (1) to strategy (4). In short, easier strategy had larger coding value and more difficult strategy had smaller coding value. The negative coefficients indicated that the strategy condition with larger coding values (an easier strategy condition) corresponded to a smaller intercept (shorter response time), whereas the strategy condition with smaller coding values (a more difficult strategy condition) corresponded to a larger intercept (longer response time). As the students moved from strategy (1) (e.g., strategy with high schema automaticity and low WML) to strategy (4) (e.g., strategy with low schema automaticity and high WML), the intercept increased. It supported our hypothesis that the strategy with high schema automaticity and low WML would be associated with shorter response time, supporting Hypothesis 2.

At Level 3 (student-level), the independent variable was phonological loop and the dependent variable was the intercept of Level 2. The phonological loop (*γ_001_* = -0.1017) negatively predicted response time on multi-step response time. As the phonological loop skill increased, the portion of intercept at Level 2 that was determined by phonological loop decreased. The higher the score on phonological loop, the lower the score on response time (shorter response time), supporting Hypothesis 3.

## Study 2

DeStefano and LeFevre (2004) recommended that in order to further understand the role of working memory in arithmetic, researchers should systematically manipulate factors such as problem conditions, problem complexity, task requirement, and so on. Thus, it is important to manipulate task characteristics through different approaches to examine whether similar findings regarding automaticity and WML could hold true. In Study 2, the task characteristics were manipulated through the levels of schema automaticity by using one-time versus two-time regrouping and through the WML by using partial versus complete decomposition. The level of schema automaticity was manipulated through regrouping. Regrouping is defined as making groups of 10s when adding two numbers and is another name for carrying (Green et al., 2007). High schema automaticity is defined as one-time regrouping and low schema automaticity is defined as two-time regrouping. Empirical studies showed that the number of regroupings had an impact on the difficulty level of the arithmetic problems (Imbo et al., 2007; Klein et al., 2010), which led to different levels of automatic retrieval (Siegler and Shrager, 1984; Ashcraft, 1992; Ashcraft and Christy, 1995; Hoyer et al., 2003). Problems with one-time regrouping corresponded to higher levels of schema automaticity whereas problems within two-time regrouping corresponded to lower levels of schema automaticity. The WML was manipulated through complete decomposition or partial decomposition, which led to a different number of steps in problem solving (Lemaire and Callies, 2009). In partial decomposition, only one operand was decomposed, so WML was low. In complete decomposition, two operands were both decomposed, so WML was high. Thus, we systematically manipulated the difficulty levels of automaticity and WML using different arithmetic approaches in Study 2. A similar three-level HLM analysis was utilized. If the findings in Study 1 would hold true in Study 2, it would enhance the generalization of the findings regarding the roles of automaticity and WML in mental arithmetic in Chinese students.

### Design

The dependent variable was multi-step mental addition performance. The independent variables included single-step mental addition performance, strategy use (we manipulated the levels of schema automaticity and WML to reflect four strategy conditions), and phonological loop. To account for student-level, strategy-level, and item-level variances, a three-level HLM analysis was applied. At the item level (Level 1), we used single-step mental addition as the independent variable and multi-step mental addition as the dependent variable. At the strategy level (Level 2), we used the four strategy conditions as the independent variable and the intercept of Level 1 as the dependent variable. At the student level (Level 3), we used the phonological loop as the independent variable and the intercept of Level 2 as the dependent variable.

### Measures and Procedures

#### Strategy

Similar to the design used in Study 1, we alternated two aspects of the structural features of addition problems: Schema automaticity was manipulated by the steps of regrouping involved in operations (i.e., one-time regrouping indicates high schema automaticity and two-time regrouping indicates low schema automaticity) and WML was manipulated by whether the addition involved partial decomposition (low WML) or full decomposition (high WML). There were four strategy conditions for each original question (e.g., 29 + 14 =), consisting of (1) problems with high schema automaticity and low WML such as (29 + 10) + 4 =? (one-time regrouping and partial decomposition), (2) problems with high schema automaticity and high WML such as (10 + 10) + (9 + 4) =? (one-time and complete decomposition), (3) problems with low schema automaticity and low WML such as (29 + 8) + 6 =? (two-time regrouping and partial decomposition), and (4) problems with low schema automaticity and high WML such as (13 + 9) + (16 + 5) =? (two-time regrouping and full decomposition). See examples in Table 4.

**Table 4:** Addition problems used during simultaneous presentation and descriptive statistics (Study 2).

Original problems	High automaticity		Low automaticity	
	Low WML (1)	High WML (2)	Low WML (3)	High WML (4)
29 + 14 = 43	(29 + 10) + 4 =	(10 + 10) + (9 + 4) =	(29 + 8) + 6 =	(13 + 9) + (16 + 5) =
18 + 34 = 52	(18 + 30) + 4 =	(10 + 30) + (8 + 4) =	(18 + 26) + 8 =	(12 + 25) + (6 + 9) =
12 + 49 = 61	(10 + 49) + 2 =	(10 + 40) + (2 + 9) =	(8 + 49) + 4 =	(4 + 23) + (8 + 26) =
14 + 49 = 63	(10 + 49) + 4 =	(10 + 40) + (4 + 9) =	(6 + 49) + 8 =	(6 + 25) + (8 + 24) =
43 + 28 = 71	(40 + 28) + 3 =	(40 + 20) + (3 + 8) =	(17 + 28) + 26 =	(26 + 7) + (17 + 21) =
63 + 18 = 81	(63 + 10) + 8 =	(60 + 10) + (3 + 8) =	(24 + 18) + 39 =	(29 + 16) + (34 + 2) =
23 + 59 = 82	(20 + 59) + 3 =	(20 + 50) + (3 + 9) =	(16 + 59) + 7 =	(17 + 34) + (6 + 25) =
57 + 34 = 91	(57 + 30) + 4 =	(50 + 30) + (7 + 4) =	(57 + 16) + 18 =	(14 + 28) + (43 + 6) =
RT M/SD in seconds	5.73 (2.45)	6.02 (2.61)	11.75 (6.53)	20.30 (10.53)
Cronbach’s α for RT	0.83	0.85	0.82	0.87


*WML, working memory load. (1), (2), (3), and (4), conditions (1), (2), (3), and (4). Condition 1: one-time regrouping and partial decomposition. Condition 2: one-time regrouping and complete decomposition. Condition 3: two-time regrouping and partial decomposition. Condition 4: two-time regrouping and complete decomposition. RT, response time.*

Our four strategy conditions were nominal scale variables that included four categories of strategies. Thus, dummy variables were created to analyze categorical variables. First, we treated strategy condition (1) as one category and the remaining three conditions as another category. Then, we had the coding for strategy-a (3, -1, -1, -1). Second, among the strategy conditions (2), (3), and (4), we treated condition (2) as one category, and conditions (3) and (4) as another category. Then, we obtained strategy-b (0, 2, -1, -1). Finally, we compared conditions (3) and (4), so we obtained strategy-c (0, 0, 1, -1). We did not need a fourth dummy variable to represent condition (4) because all four strategy conditions were mutually exclusive (they did not overlap) and exhaustive (no other levels exited for this variable; Ding, 2000).

#### Multi-Step Addition Problems (Simultaneous Presentation)

First, we selected eight addition problems (the range of sums was 43 to 91, *M* = 68, *SD* = 15.48). The eight problems were designed following four rules: (a) within half of the problems, the larger operands were in the left position (e.g., 63 + 18 =); within the other half of the problems, the larger operands were in the right position (e.g., 12 + 49 =); (b) the digits were not repeated in the same unit or place value across operands (e.g., 64 + 14); (c) no digits were repeated within operands (e.g., 55 + 11); and (d) no operand had 0 in the ones place value (Lemaire and Callies, 2009).

By alternating the levels of automaticity and WML, there were four conditions for eight original problems. Thus, we had 32 problems in total. Table 4 presents how we alternated schema automaticity and WML in the four testing conditions. Cronbach’s α was 0.92 for response time and 0.67 for accuracy, which is acceptable (DeVellis, 1991). E-prime was used for programming. The details of the procedure were similar to the description in Study 1.

#### Single-Step Addition Problems

The same 32 addition problems were re-used. However, to obtain accuracy and response time on single-step addition, we decomposed the multi-step addition problems and generated 77 single-step addition problems. For example, (29 + 10) + 4 = would be decomposed to two single-step addition problems, including 29 + 10 = and 39 + 4 =. Some decomposition of the multi-step addition problems would lead to repeated single-step addition problems, and we only retained one of them. All problems were presented randomly by the computer. The stimulus of “+” was flashing in the center of the computer screen and it continued flashing for 150 ms. Then, the single-step addition problem was presented. The examinee orally reported the answer, and the examiner manually entered the answer and pushed “enter” for the next item to be presented. After the examinee completed 20 items in a row, the examinee took a short break. The computer automatically recorded the accuracy and response time (i.e., the duration was from the point of stimulus presentation to the point that the examiner hit the enter key) for each item (i.e., both correct or incorrect items). We considered the mean response time of all single-step addition problems involved in a multi-step mental addition as the single-step response time corresponding to that multi-step mental addition response time.

#### Working Memory Measure (Phonological Loop Task)

The details of the phonological loop task were provided in Study 1.

To counter an order effect, all problems of each task were randomly presented by the computers. E-prime was used for programming. Prior to testing, the participants received training through practice items. The participants completed three tasks in a random order.

### Participants

Running a power analysis on a repeated measures ANOVA with four measures, a power of 0.80, an alpha level of 0.05, and a medium effect size (*f* = 0.25) requires a sample size of at least 24 (Faul et al., 2013). We recruited 43 typically developing fourth graders (female = 25, male = 18) who should have fluently mastered under-100 addition, subtraction, multiplication, and division by the time of testing. They ranged from 9 to 11 years old (*M* = 9.42, *SD* = 0.79), with 22 females and 21 males. The participants were randomly recruited from an elementary school in China. All children did not carry documented disabilities and did not receive training on mental arithmetic. This study was approved by the Research Ethics Committee of Beijing Normal University and the principals of the participating schools. Written and Informed consent was obtained from the parents/legal guardians of participants.

### Results and Discussion

In Study 2, the main goal was to examine how single-step mental addition, strategy use, and working memory measure affected multi-step mental addition. A three-level HLM analysis was applied to account for student-level, strategy-level, and item-level variances. The Level 3 sample size was 43, the Level 2 sample size was 172 (43 students completed four strategy conditions), and the Level 1 sample size was 1,375 (43 students completed 32 items and there were missing items). Based on Chang (2003, 2004), we maintained four decimals in the HLM analysis.

All of the participants had very high levels of accuracy (89.24% for all conditions, *SD* = 7.8%) and there was little variation among the participants. Thus, the measure of accuracy was excluded as a variable for analysis. We only used participants’ correct response time for further analysis. The descriptive statistics for different variables are listed in Table 5.

**Table 5:** Descriptive statistics of response time at item-, strategy-, and student-level (Study 2).

Variables	*N*	Mean	*SD*	Min	Max
**Item-level (Level 1)**					
Multi-step RT	1224	9.98	7.73	2.50	66.22
Single-step RT	1224	2.80	1.32	0.94	10.87
**Strategy-level (Level 2)**					
Strategy-a	171				
Strategy-b	171				
Strategy-c	171				
**Student-level (Level 3)**					
Phono	43	40.14	9.18	7.00	48.00


*We only analyzed correct response time. RT, response time (measured by seconds). There were 32 testing items. The multi-step response time was calculated based on the response time on each testing item. The single-step response time was calculated based on the response time on each testing item divided by the presentation steps involved in that item. Phono, phonological loop.*

In Table 6, the dependent variable was the average response time of multi-step addition at Level 1 (item level). The independent variable was the response time of single-step addition at Level 1, indicating simple addition skills. *γ_100_* (1.5751) was significant and suggested that the single-step response time had an effect on multi-step response time in the positive direction. It indicated that better (faster) response time on single-step mental addition decreased the response time on multi-step mental addition, supporting Hypothesis 1.

**Table 6:** Effects of automaticity, strategy, and phonological loop on response time: three-level regression coefficients (Study 2).

Fixed effect		Coefficient	*SE*	T-ratio	Approx. df	*P*
**Multi-step RT as the outcome measure**						
*Student-level (Level 3)*							
For INTRCPT1	π_0_					
INTRCPT2	β_00_					
INTRCPT3	γ_000_	8.2621	1.6268	5.08	41	<0.001
Phono	γ_001_	–0.0530	0.0257	–2.06	41	0.045
*Strategy-level (Level 2)*							
For STATEGY-A	β_01_					
INTRCPT3	γ_010_	–1.3622	0.0925	–14.73	125	<0.001
For STATEGY-B	β_02_					
INTRCPT3	γ_020_	–2.2642	0.1893	–11.96	125	<0.001
For STATEGY-C	β_03_					
INTRCPT3	γ_030_	–4.1992	0.3914	–10.73	125	<0.001
*Item-level (Level 1)*							
For ST-RT slope	π_1_					
INTRCPT2	β_10_					
INTRCPT3	γ_100_	1.5751	0.3050	5.16	1009	<0.001


*We only analyzed correct response time. Phono, phonological loop; RT, response time; ST-RT, single-step response time; INTRCPT, intercept. It is common to retain four decimals for HLM results (Chang, 2003).*

At Level 2 (strategy-level), the dependent variable was the average response time of multi-step mental addition. Four strategy conditions were treated as dummy variables, including strategy-a, strategy-b, and strategy-c. *γ_010_* (-1.3622), *γ_020_* (-2.2642), and *γ_030_* (-4.1992) were all statistically significant, suggesting that strategy use had effects on multi-step response time in the negative direction. The negative coefficients indicated that the strategy condition with larger coding values (easier strategy condition) corresponded to a smaller intercept, whereas the strategy condition with smaller coding values (more difficult strategy condition) corresponded to a larger intercept. In other word, as students moved from strategy (1) (easier strategy) to strategy (4) (more difficult strategy), the intercept determined by the strategies increased (indicating longer response time). This supported our Hypothesis 2 that the strategy with high schema automaticity and low WML would be associated with a shorter response time.

At Level 3 (student-level), the phonological loop was the independent variable and the intercept of Level 2 was the dependent variable. The phonological loop (*γ_001_* = -0.0530) negatively predicted response time on multi-step mental addition. As the phonological loop skill increased, the portion of intercept of Level 2 determined by phonological loop decreased. This finding supported our Hypothesis 3 that the higher the score of the phonological loop, the shorter the response time.

## General Discussion

### Main Findings

The findings reveal the important roles of working memory, single-step mental addition skills, and strategy use in multi-step mental addition. We manipulated the difficulty levels of the tasks through the dimension of WML and schema automaticity by using different approaches in Study 1 and Study 2. There are three main findings revealed in Study 1 and Study 2. First, children’s shorter response time on single-step mental addition was associated with shorter response time on multi-step mental addition, regardless of how we manipulated the levels of WML and schema automaticity. Second, different strategy use was enforced through the four strategy conditions for which we manipulated the difficulty levels of schema automaticity and WML. Easier strategy was associated with shorter response time. Third, stronger phonological loop was associated with shorter response time on multi-step mental addition.

Single-step response time was considered as children’s fluency on simple addition facts. Our findings support the importance of fluency in single-step addition facts in order for children to perform efficiently on multi-step mental addition. These findings confirm the importance of fluency in basic arithmetic facts, which is consistent with previous findings indicating that direct retrieval of simple mathematic facts is the most advanced and most efficient strategy with regard to problem solving speed and accuracy (Siegler, 1988; Geary et al., 2004). According to the cognitive load theory (Sweller, 1988; Paas et al., 2003; van Merriënboer and Sweller, 2005), high fluency on single-step addition largely reduces the load on working memory, freeing up working memory for more complex operations such as multi-step addition. Low fluency on single-step addition facts indicates that children who do not directly retrieve basic addition facts from their unlimited long-term memory could be overwhelmed by the number of interactive single-step addition facts that need to be processed simultaneously before multi-step addition can be processed (Paas et al., 2010). In the case that children are not fluent with single-step addition facts, their execution of single-step addition requires substantial resources of working memory in order to consciously process the intermediate sums of single-step addition. Cumulatively, the process to execute the intermediate sums of single-step addition, memorize the intermediate sums, and add all intermediate sums to form the total sums would warrant a large amount of processing time (longer response time).

In Ding et al. (unpublished), we found that student response time followed the order of strategy (1) < strategy (2) < strategy (3) < strategy (4), from the fastest condition to the slowest condition, by examining the descriptive statistics. The findings of the HLM analysis concurred with our previous observations (Ding et al., 2017), suggesting that high schema automaticity and low WML corresponded with higher accuracy rate and shorter response time. Under the strategy (1) condition, the problems were presented with high schema automaticity and low WML such as 8 + 12 + 6 (i.e., the difficulty levels on both dimensions were low). The problem has an intermediate sum of 10 and only has two steps. Thus, strategy (1) yielded the fastest response time. Under the strategy (4) condition, the problems were presented with low schema automaticity and high WML such as 8 + 6 + 3 + 7 + 12 (i.e., the difficulty levels on both dimensions were high). Thus, strategy (4) yielded the slowest response time. Under strategy (2), problems with high schema automaticity and high WML (8 + 2 + 7 + 3 + 6 =), and strategy (3) problems with low schema automaticity and low WML (8 + 6 + 12 =), only one dimension of the problem was difficult and the other dimension of the problem was easy. The findings in Ding et al. (2017) indicated that sacrificing resources on WML while performing easier tasks (i.e., tasks that students could retrieve automatically) rendered shorter response time, whereas less demand on WML did not compensate for the limits of automaticity, suggesting that children performed better in condition (2) than they did in condition (3). Our Level-2 HLM analysis supported our previous observations (Ding et al., 2017). All coefficients at Level 2 are negatively significant, indicating that an easier strategy condition (condition with larger dummy variable coding value) led to a smaller intercept determined by that strategy (i.e., shorter response time). In other words, the use of a more effective strategy led to shorter response time on multi-step mental addition.

Children’s performance on the phonological loop task negatively predicted the response time on multi-step mental addition, concurring with Liu et al. (2017), Ding et al. (unpublished). Higher phonological loop scores corresponded to shorter response time on multi-step mental addition. The findings underline the important role of phonological loop in mental arithmetic in Chinese children. Although there have been mixed findings regarding the role of phonological loop in single-step mental arithmetic in empirical studies conducted with Western participants (e.g., Lemaire et al., 1996; De Rammelaere et al., 1999, 2001; Seitz and Schumann-Hengsteler, 2000, 2002; Hecht, 2002), the critical role of phonological loop has been demonstrated in single-step mental arithmetic in Korean participants (Lee and Kang, 2002) and in multi-step mental arithmetic in Chinese participants (Liu et al., 2017; Ding et al. unpublished). We attributed such a universal role of phonological loop in mental arithmetic to the unique mathematics instructional approach adopted in the Chinese education system. The Chinese school system emphasizes practice and drills on basic mathematic facts. A large amount of class time is designed to enhance children’s fluency on simple arithmetic such as addition, subtraction, multiplication, and division. For example, children are required to rote memorize multiplication tables from 1 × 1 to 9 × 9, and children often memorize such arithmetic facts through verbal rehearsal (e.g., one one equals one, one two equals two). For one-digit or two-digit addition and subtraction, rote memorization is also greatly encouraged. Thus, it is rare to observe Chinese children attempting a wide range of strategies to tackle simple arithmetic problems because they often rely on verbal modality to directly retrieve the results from long-term memory. China’s Compulsory Education Law (National People’s Congress, 1986) is responsible for students ranging from Kindergarten to Grade 9, and students within the age/grade range are entitled to free public education. According to the data released by the Ministry of Education (2014), there were 254,000 public schools serving students from Kindergarten to Grade 9, whereas there were only 10,425 private schools serving K-9 students (only 4% of the K-9 schools are private). In China, the standard mathematics curriculum is developed by the Ministry of Education to avoid disparities in education caused by regional differences, and all public schools (96% of all K-9 schools) utilize the standard mathematics curriculum mandated by the central government. In other words, there is very little variation in terms of how Chinese children are taught basic mathematic facts. Early mathematics teaching in China encourages language-specific representations of basic mathematic facts, which supports the critical role of phonological loop in our findings.

It is ideal to analyze findings from the aspects of accuracy and response time. However, it is noteworthy that Chinese children were fairly accurate on mental addition (91.5% accuracy rate for Study 1 and 89.24% accuracy rate for Study 2), regardless of how the testing conditions were manipulated. Thus, we did not include accuracy in the final analysis due to little variation among the students (i.e., students were fairly accurate regardless whether they spent more or less time on problems). The findings concurred with the high accuracy rate of Chinese children reported in Ding et al. (unpublished). In the present studies, we artificially increased the difficulty levels of the strategy conditions (i.e., strategies 1, 2, 3, and 4), and the complexity of the problem formats appeared to affect the response time (i.e., children took longer to respond to more complex problems). However, the Chinese children in our studies continued to accurately execute the problems and provide correct answers, regardless of the increased steps or decreased schema automaticity to retrieve arithmetic facts. The increased difficulty levels of the problems obviously sacrificed their response time, but not their accuracy rate. In China’s elementary mathematics curriculum (People’s Education Press, 2017), exact arithmetic calculation is largely emphasized, with less emphasis on number estimation (i.e., teachers discourage approximate answers or guessing but encourage accurate answers). A large amount of homework and in-class practice serve to enhance children’s calculation accuracy. Our findings echoed the evidence of performance advances for East Asian students in simple arithmetic that occur in elementary school as early as Kindergarten (Siegler and Mu, 2008), secondary school (Stevenson et al., 1993), and beyond (Stevenson and Stigler, 1992).

We used different approaches to alternate the difficulty levels of the strategies in Study 1 and Study 2. In Study 1, we alternated the task difficulty levels through the dimension of WML (i.e., two steps versus four steps) and the dimension of schema automaticity (i.e., intermediate sums were 10 or were not 10; Lemaire and Callies, 2009; Klein et al., 2010). Study 1 extended our previous study (Ding et al., 2017) in mental multiplication to mental addition, but followed the same design for task development. In Study 2, the schema automaticity was manipulated by the steps of regrouping involved in operations (i.e., one-time regrouping versus two-time regrouping), and WML was measured by whether the addition involved partial decomposition (low WML) or full decomposition (high WML; Lemaire and Callies, 2009), which was not utilized in previous studies. The general findings in Study 1 held true in Study 2, even though the strategy conditions were manipulated differently.

## Limitations and Conclusion

We note that our studies have shortcomings. First, the participants were limited to two independent samples of third graders and fourth graders in large cities of China. The findings might not be generalizable to learning of arithmetic in other countries due to possible differences in instructional approaches and learner characteristics. Second, we assumed that if a problem was presented in a specific way (e.g., imposed problem format such as 8 + 12 + 6, then children would calculate 8 + 12 = 20 first and then calculate 20 + 6 = 26 in that order); that is, children would solve problems according to the enforced problem format. It remains unclear whether a small portion of participants might have generated their own strategy (e.g., 8 + 12 + 6, then children would calculate 12 + 6 = 18 first and then calculate 8 + 18 = 26), regardless of the problem format we enforced. There was no mechanism to prevent spontaneous strategy use that did not follow the imposed problem format. Nevertheless, even if in some cases the students used some strategies to reorganize the sequence of calculating a problem, they must have spent some time observing the digital features of the problem and then making decisions on what strategies they could generate and use, which would have led to increased response time. Third, the measures of accuracy and response time should be used for analysis in an ideal situation. For example, both analyses for accuracy and response time were provided for the examination of mental multiplication in Ding et al. (2017). However, in the samples in the present study, students were fairly accurate on all addition task conditions regardless of how we manipulated the tasks, although they demonstrated differentiated response time under different addition task conditions. Due to the little variance of accuracy rates among the participants, the measure of accuracy rate was excluded for final analysis.

Despite the shortcomings, the present studies extend the literature in a number of ways. First, we extended our alternation of WML and automaticity from mental multiplication (Ding et al., 2017) to mental addition. Second, within mental addition, we applied different approaches to alternate the difficulty levels of WML and schema automaticity in Study 1 and Study 2, and the general findings were consistent in both studies. Our findings indicate that future researchers might consider utilizing different approaches to alternate WML and schema automaticity and examine whether the findings hold true under different testing conditions. Third, the present studies underscore the importance of enhancing children’s fluency in simple arithmetic, the use of effective strategy, and the important role of verbal representation of arithmetic facts in Chinese children. The homogenous evidence supports the activation of phonological loop during mental arithmetic problem solving in Chinese children. It highlights the importance of evaluating the linguistic features and instructional contexts in which children become fluent with basic arithmetic facts.

## Author Contributions

YD and RL designed the study and wrote the manuscript together. YD was in charge of the submission and review process. HL assisted for data analysis and interpretation. JW helped for data collection and data analysis. RZ and RJ assisted for editing and checking the results.

## Conflict of Interest Statement

The authors declare that the research was conducted in the absence of any commercial or financial relationships that could be construed as a potential conflict of interest.
